# Assessing the Correlation Between Anthropometric Measurements and Bone Densitometry As Indicators of Bone Health in Adult Women in the Community

**DOI:** 10.7759/cureus.68162

**Published:** 2024-08-29

**Authors:** Rohan Chandanwale, Kshitija Chandanwale, Rutuja Chandanwale, Ajay Chandanwale

**Affiliations:** 1 Orthopaedics, Jawaharlal Nehru Medical College, Datta Meghe Institute of Medical Sciences, Wardha, IND; 2 Surgery, Maharashtra Post Graduate Institute of Medical Education and Research, Nashik, IND; 3 Dermatology, Mahatma Gandhi Medical College and Research Institute, Mumbai, IND; 4 Orthopaedics, Directorate of Medical Education and Research, Mumbai, IND

**Keywords:** quantitative ultrasound, anthropometric measurements, menopause, bone mineral density, osteoporosis

## Abstract

Introduction

Osteoporosis is one of the most prevalent bone diseases in humans and is a significant global public health issue since it is a risk factor for age-related fractures. Fracture risk is significantly influenced by bone mineral density (BMD). Recent research has revealed that there are various genetic and environmental variables that are similar between obesity and osteoporosis. The relationship between anthropometric measurements including weight, body surface area, height, and fat mass and BMD has been the subject of several studies. Decreased bone mass and a high risk of fracture have been linked to low BMI.

Materials and methods

A total of 370 female patients were included in this study. Anthropometric measures, such as weight and height, were taken in accordance with international standards. We measured the T-score, Speed of Sound (SOS) (in metres/s), Broadband Ultrasound Attenuation (BUA), and Stiffness Index (SI) of the participants using a portable quantitative ultrasonic bone densitometer with a gel-coupled system in a temperature-controlled environment (26 ± 1°C) to estimate the BMD. In this study, we analyzed the relationships between anthropometric measurements such as weight, height, BMI, and waist-hip ratio (WHR) and BMD.

Results

In our study, we found that the population falling under the categories of Underweight and Obese have shown to have reduced BMD. There is an association between normal BMI and normal BMD. BMI can considerably affect one’s risk of developing osteoporosis. Therefore, BMI and weight can be used to screen those who are at risk of having osteoporosis and its associated problems. We also observed an association between menopause and BMD measured by Quantitative Ultrasound (QUS). In the study population, post-menopausal women had a 4 times higher risk of osteoporosis than pre-menopausal women (OR = 4.46).

Conclusion

Calcaneal QUS is potentially helpful as a pre-screening tool for the evaluation of osteoporosis, although it must be based on device-specific cut-offs that have been tested in the populations for which they are intended to be used in a pre-screen or stratification methodology.

## Introduction

Reduced bone mineral density (BMD) and deteriorating bone microarchitecture are the hallmarks of osteoporosis, a bone metabolic condition that increases skeletal fragility and fracture risk [1]. Due to an increase in population life expectancy, osteoporosis prevalence, and the large number of fractures caused by morbidity and mortality, osteoporosis is one of the most prevalent bone diseases in humans and is a major global public health issue. Fracture risk is significantly influenced by BMD [2]. According to a prior study, osteoporotic fractures cost the United States $13 billion in 1995 [3]. As we entered the twenty-first century, it was inevitable that this burden would rise due to an increase in life expectancy. Even in the most industrialized countries, the prevalence of overweight and obesity is rising despite public health initiatives [4].

The relationship between anthropometric measurements including weight, body surface area (BSA), height, and fat mass, and BMD has been the subject of several studies. Decreased bone density and a high fracture risk have been linked to low BMI. It has also been demonstrated that a higher calcium absorption rate is favorably correlated with body height. BSA is subsequently associated with osteoporotic fractures, although BMI is not [5]. BSA is a non-linear mix of weight and height. Additionally, it has been suggested that certain anthropometric characteristics, such as the patient's weight, may be used to improve the diagnostic utility of BMD in female patients at risk for osteoporotic fractures [6]. Recent research has revealed that there are various genetic and environmental variables that are similar between obesity and osteoporosis. Cardiovascular and metabolic issues also affect obese osteoporotic patients more frequently than individuals with normal body mass indices [7]. On the other hand, weight loss plans and operations put the health of overweight people at risk due to calcium malabsorption leading to osteoporosis. This is especially crucial when discussions regarding weight loss in postmenopausal women are involved [8].

Previous studies have shown that, to prevent bone loss, weight reduction should be gradual and accompanied by an increase in calcium consumption. Interestingly, compared to obese women, overweight women (BMI between 25 and 29.9 kg/m2) are more likely to experience bone loss (BMI between 30 and 40). To put it another way, the relationship between anthropometric measurements and BMD is crucial not just at cross-sections but also when it changes over time, as in the case of weight reduction programs [9]. As a result, population-specific cut-off points must be used for these indices because it has been demonstrated that various ethnic groups may have varied anthropometric measurements [10]. In addition to advanced age, white and oriental races, chronic estrogen deficiency, and genetic factors (family history of fracture and osteoporosis in first-degree relatives), all of these factors are unchangeable and are among the predictors of BMD. In reality, however, there are controllable variables, including body composition, sedentary behavior, smoking, chronic corticosteroid treatment, excessive alcohol and coffee consumption, and inadequate sunshine exposure [11]. Lower body mass is implicated in fragility fractures, in which case bone density is the key quantifiable risk factor. A BMI is linked to a much higher risk of fractures [12]. South India is thought to have an osteoporosis prevalence that ranges from 20% to 50% based on the cohort that was examined [13].

As a result, identifying the disease through community or high-risk group screening may aid in the prevention of fragility fractures. There have been several described screening techniques. Out of these, dual-energy X-ray absorptiometry (DXA) measures of BMD serve as the foundation for therapy in the majority of cases. Its availability and cost, however, prevent broad usage, hampering efforts to detect and treat osteoporosis. The link between anthropometric measurements including body weight, BMI, waist circumference, hip circumference, and BMD has been demonstrated in several studies [14]. Some researchers have proposed anthropometric-based risk prediction algorithms. These would likely help in screening and detecting high-risk patients who might then be referred, even if they wouldn't replace DXA and BMD values [15]. In order to apply anthropometric indices in a population, cut-off values based on ethnicity are required. Few people have looked at the relationship between anthropometric measures and BMD, and it is currently unexplored in India. In this study, a sample of adult women who were receiving bone densitometry at a tertiary care hospital in Wardha were examined to determine any relationships between anthropometric measurements such as weight, height, BMI, and waist-hip ratio (WHR) and BMD.

## Materials and methods

Type of study design

This is a cross-sectional study.

Sample size

Considering a prevalence of 43% and using a z-value of 1.962 at a 5% type I error, the sample size was calculated using the following formula: n = p × (1 - p ) × z^2^/E^2^, where, n: sample size p: sample proportion, z: found by using a z-score table, E: margin of error (to be divided to get a decimal).

Substitute the inputs and round up to the nearest whole number:

n = 0.43 (1-0.43) x 1.962² / 0.05²

n = 0.43 x 0.57 x 3.84 / 0.0025

n = 0.941184/0.0025

n = 376.47 rounded up to

n = 370 

Objectives

The objectives of the study are to examine calcaneal BMD using quantitative ultrasound in adult females of the study population, and to assess various anthropometric measurements as indicators of bone health among adult women. Additionally, the study aims to correlate values of bone mineral density on quantitative ultrasound (QUS) with anthropometry in adult females, and to detect osteoporosis among pre-menopausal and post-menopausal adult women.

Inclusion criteria

All adult women aged 18 years and above in the community who were willing to participate were included in the study.

Exclusion criteria

The female population under the age of 18 years and adult females not willing to participate were excluded from the study.

A total of 370 female patients were enrolled in this study. Anthropometric measures such as weight and height were taken in accordance with international standards by professional technicians while the subjects were wearing light clothes and without shoes [16]. Each anthropometric measurement was performed using the same method and tool. All measures underwent rigorous quality control checks. Height was measured to the nearest 0.1 cm using a wall-mounted stadiometer that took readings in 1-mm steps, and weight was measured to the nearest 0.1 kg using a portable digital scale. The BMI was computed by dividing the body weight in kilograms by the square of the height in meters (kg/m^2^). Waist and hip circumferences were measured with a nonelastic tape while the subjects were standing; the WHR was computed by dividing the waist circumference by the hip circumference. Assessment of the prevalence of both general and abdominal obesity was conducted using the WHO classification of BMI, identifying the BMI status of the women as follows: underweight (BMI < 18.5), normal (18.5 ≤ BMI ≤ 24.9), overweight (25 ≤ BMI ≤ 29.9), and obese (30 ≤ BMI).

Assessment of BMD

**Figure 1:**
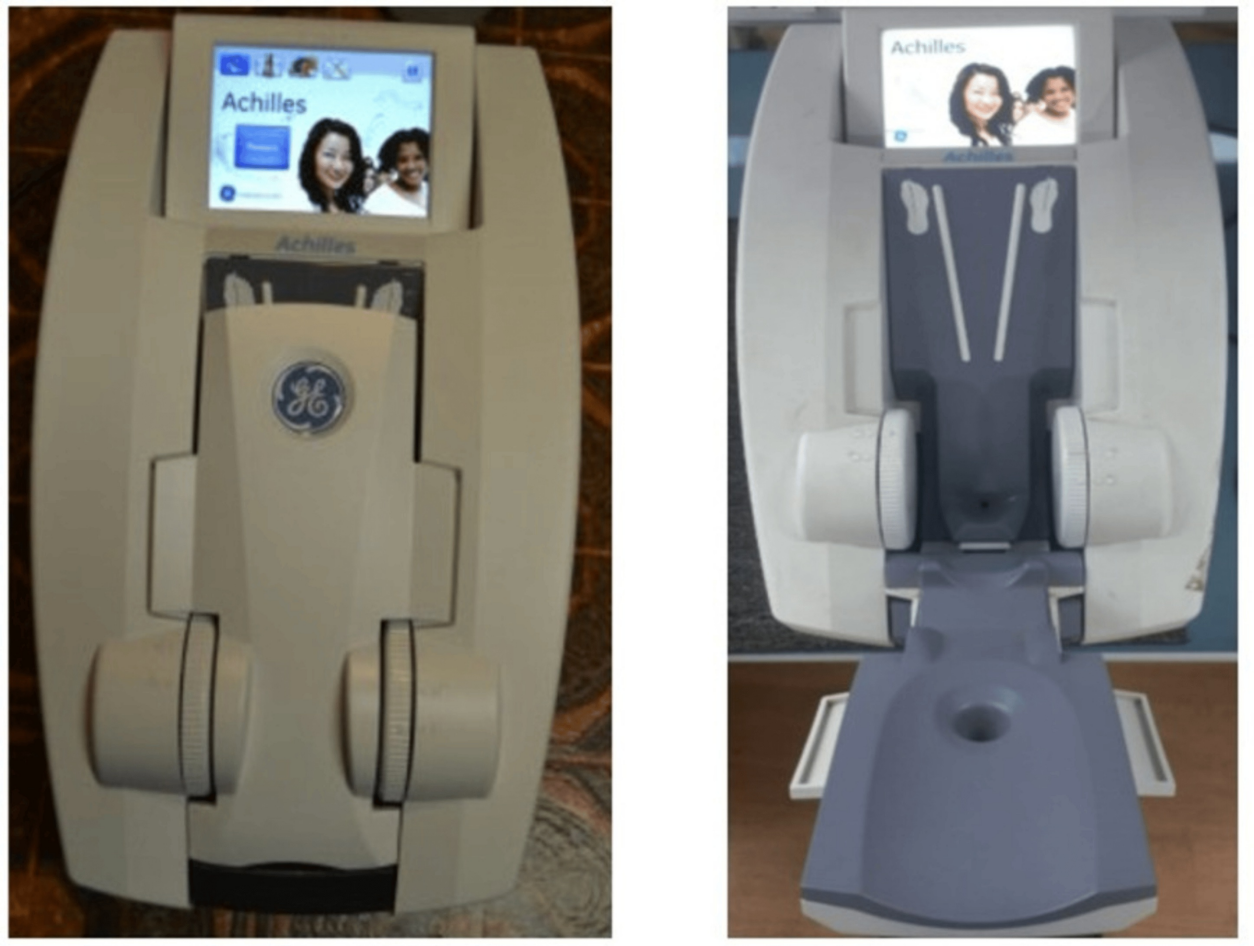
Achilles EXP-II, GE Medical Systems (CHINA) & Co., Ltd.

At the right calcaneus, the Speed of Sound (SOS) (in m/s) was measured. While Dual-energy X-ray Absorptiometry (DXA) of the central skeleton is considered the "gold standard" for BMD measurement, unfortunately, the requisite equipment is not easily accessible at the community level [17]. QUS technology for calculating BMD has been increasingly regarded as a diagnostic method for determining osteoporosis. Therefore, we measured the T-score, SOS (in meters/s), Broadband Ultrasound Attenuation (BUA), and Stiffness Index (SI) of the participants using a portable quantitative ultrasonic bone densitometer with a gel-coupled system (Achilles EXP-II, GE Medical Systems (CHINA) & Co., Ltd) in a temperature-regulated environment (26± 1°C) to estimate the BMD as shown in Figure 1.

**Figure 2:**
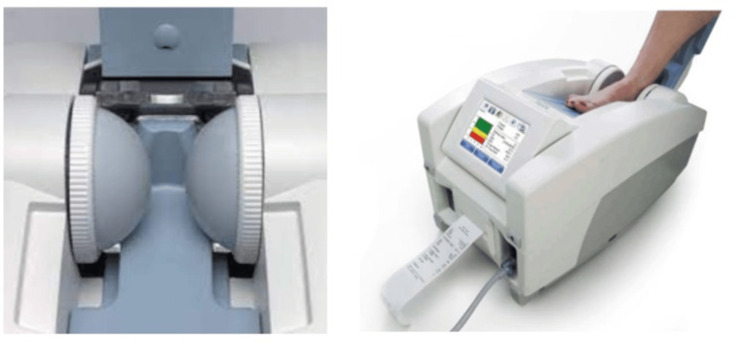
Inflated membranes used to transmit ultrasound waves.

The Achilles test uses a membrane filled with warm water, which is placed against the heel to provide a passageway for the ultrasound waves. Warm water enclosed between inflated membranes surrounds the heel. Isopropyl alcohol is employed to create a connection between the membranes and the heel. The membrane is white in hue. It takes approximately ten seconds to perform one measurement, as shown in Figure 2. A higher value is related to a decreased risk of osteoporosis.

Additionally, the device's performance was evaluated against DXA. The BMD of the calcaneus and lumbar spine were also linked with the SOS as determined by the CM-100 (r=0.77 and r=0.65, respectively). There were no discernible differences in the areas under the ROC curves for SOS determined by the Achilles EXP-II and calcaneus BMD determined by DXA in predicting osteoporosis determined by the lumbar spine BMD determined by DXA. Each survey day’s first measurement was preceded by a quality assurance phantom scan to ensure that the equipment was calibrated. The SOS is determined by dividing the length of the body part under study by the transmission duration of the sound waves, measured in meters per second (m/s). The term BUA refers to the slope between the attenuation of sound signals and their frequency, measured in decibels per megahertz (dB/MHz). Attenuation occurs as sound waves pass through soft tissue and bone because the energy is absorbed.

Statistical analysis

Age-related groupings were used to stratify all statistical analyses. Continuous data are presented as mean (SD), while categorical data are reported as number (percentage). The mean difference between continuous variables was evaluated using a t-test. Categorical variables were compared using the Chi-square test. The correlation between continuous variables was evaluated using Pearson's correlation test. The link between anthropometric measurements and BMD was examined using the correlation coefficient. Statistics were deemed significant at p < 0.05. The statistical analysis was performed using Epi-info 7 software.

## Results

As anthropological parameters were analyzed, one of the parameters was BMI. Table 1 shows that most of the study participants, 213 (58%), had a normal BMI (18.5-24.9), followed by the underweight group, 85 (23%) with a BMI < 18.5. This was followed by pre-obese (BMI 25-29.9) women, who constituted 57 (15%) of the study subjects. The obese class 1 (BMI 30-34.9) and obesity class 2 (BMI >35) were 13 (3%) and 2 (1%), respectively.

**Table 1:** BMI categories among the study population.

BMI	Category	No. (%)
<18.5	Underweight	85 (23)
18.5-24.9	Normal	213 (58)
25-29.9	Pre-obese	57 (15)
30-34.9	Obese Class 1	13 (3)
>35	Obese Class 2	2 (1)

Table 2 shows the average values of anthropometric parameters included in the study of pre- and post-menopausal women.

**Table 2:** Anthropometric characteristics of study groups in pre- and post-menopausal women.

	Post-menopausal (n_1_=200)	Pre-menopausal (n_2_=170)	t statistics, P-value
Mean age	57.21+6.64	39.19+6.68	24.92, <0.001
Height	1.62+0.07	1.61+0.06	1.46, 0.14
Weight	55.66+10.79	56.95+9.84	1.19, 0.23
BMI	21.33+4.47	21.97+3.90	1.45, 0.14
Waist circumference	100.38+2.48	98.86+2.89	5.44, <0.001
Hip circumference	102.7+1.61	101.5+2.30	5.92, <0.001

Table 3 shows that among those who had achieved menopause, the T-score on QUS was -1.17±0.74 in the age group 41-50, -1.45±1.04 in the age group 51-60, -1.87±0.91 in the group 61-70, and -2.40±0.56 in the 71-80 age group. Thus, the mean T-score on QUS decreases as age advances in the group who have achieved menopause. Similarly, in the group where menopause has not been achieved, the T-score on QUS of the 21-30 age group is -0.71±1.58, in age 31-40 is -0.66±0.90, and in the 41-50 age group is -0.74±1.03. Thus, a similar decrease in T-score on QUS has been observed as age advances in those who have not achieved menopause.

**Table 3:** T-score of the study population according to age group in pre- and post-menopausal participants. The above table shows the T-score on QUS among the study participants based on their age and menopause status. QUS: Quantitative Ultrasound.

Age	(n)	Post-menopausal		Pre-menopausal	
		Mean T score	SD	Mean T score	SD
21-30	19			-0.71	1.58
31-40	78			-0.66	0.90
41-50	118	-1.17	0.74	-0.74	1.03
51-60	97	-1.45	1.04		
61-70	54	-1.87	0.91		
71-80	4	-2.40	0.56		

Table 4 shows the association between BMI and BMD among the study participants who have achieved menopause (N=200). The mean T-score values in the various categories of BMI are observed. The mean T-score in the underweight (58) was -1.87±0.8, in the normal category was -1.32±1.05, and in the overweight was -1.26±0.94. As the BMI increases, the T-score improves in those who have menopause. There was a significant association between BMI and BMD with a significant p-value = 0.0017.

**Table 4:** Association of BMI and BMD (T-score) on QUS among post-menopausal study participants. F-statistics = 5.48, p-value = 0.0017, significant. QUS: Quantitative Ultrasound; BMD: Bone mineral density.

Group	N (count)	Mean	SD	95% CI (Mean-Lower-Upper)	
Underweight	58	-1.87	0.8	-2.04256	-1.69744
Normal	104	-1.32	1.05	-1.5242	-1.1158
Overweight+Obese	38	-1.26	0.94	-1.82449	-1.09551
Total	200				

Table 5 shows the association between BMI and BMD among the study participants who have not achieved menopause. The mean T-score values in the various categories of BMI are observed. The mean T-score in the underweight was 0.91±1.35, in the normal category was -0.62±0.93, and in the overweight was -0.60±1.04. As the BMI increases, the T-score improves in those who have not achieved menopause. There was a significant association between BMI and BMD with a significant p-value = 0.0117.

**Table 5:** Association of BMI and BMD (T-score) on QUS among pre-menopausal study participants. F-statistics = 3.77663, p-value = 0.0117. BMD: Bone mineral density; QUS: Quantitative Ultrasound.

Group	N (count)	Mean	SD	95%CI (lower limit-upper limit)	
Underweight	27	-0.91	1.35	-1.44404	-0.375959
Normal	109	-0.62	0.93	-0.796568	-0.443432
Overweight + Obese	34	-0.6	1.04	-0.995597	-0.204403
Total	170				

Table 6 shows the BUA (in dB/MHz) in the age group 41-50 is 103.64±7.90, in the age group 51-60 is 98.35±16.88, in the age group 61-70 is 95.80±11.46, and in the age group 71-80 is 90.20±3.69. As a corollary, the BUA diminishes with age following menopause. The BUA of study participants who did not reach menopause in the age group 21-30 is 111.96±24.05, in the age group 31-40 is 106.99±15.29, and in the age group 41-50 is 107.35±12.00.

**Table 6:** BUA (dB/MHz) score of the study population according to age group in pre- and post-menopausal participants. The table above depicts the age-related BUA in our study sample (N=370) based on menopausal status. BUA: Broadband Ultrasound Attenuation.

Age	(n)	Post-menopausal		Pre-menopausal	
		Mean BUA (in db/MHz)	SD	Mean BUA (in db/MHz)	SD
21-30	19			111.96	24.05
31-40	78			106.99	15.29
41-50	118	103.64	7.90	107.35	12.00
51-60	97	98.35	16.88		
61-70	54	95.80	11.46		
71-80	4	90.20	3.69		

Table 7 shows the age-wise distribution of mean SOS values in the study population. Among those who had achieved menopause, the SOS (in meters/s) was 1533.37±27.82 in the age group 41-50, 1529.70±25.68 in the age group 51-60, 1517.64±23.98 in the group 61-70, and 1510.15±21.83 in the 71-80 age group. Thus, the mean SOS decreases as the age advances in the group that has achieved menopause. Similarly, in the group where menopause has not been achieved, the mean ± SD of the 21-30 age group is 1546.47±36.26, in the age 31-40 group it is 1546.34±28.42, and in the 41-50 age group it is 1544.76±30.94. Thus, a similar decrease in the SOS has been observed as the age advances in those who have not achieved menopause.

**Table 7:** SOS of the study population according to age group in pre- and post-menopausal participants. The above table shows the SOS on QUS among the study participants based on their age and menopause status. SOS: Speed of sound; QUS: Quantitative Ultrasound.

Age	(n)	Post-menopausal		Pre-menopausal	
		Mean SOS	SD	Mean SOS	SD
21-30	19			1546.47	36.26
31-40	78			1546.34	28.42
41-50	118	1533.37	27.82	1544.76	30.94
51-60	97	1529.70	25.68		
61-70	54	1517.64	23.98		
71-80	4	1510.15	21.83		

Table 8 shows the age-wise distribution of mean SI values of the study population. Among those who had achieved menopause, the SI was 78.46±10.56 in the age group 41-50, 74.27±14.90 in the age group 51-60, 68.72±12.32 in the group 61-70, and 62.75±7.46 in the 71-80 age group. Thus, the mean SI decreases as the age advances in the group that has achieved menopause. Similarly, in the group where menopause has not been achieved, the mean ± SD of the 21-30 age group is 87.63±24.93, in age 31-40 it is 85.35±13.35, and in the 41-50 age group it has been 84.04±14.68. Thus, a similar decrease in the SI has been observed as the age advances in those who have not achieved menopause.

**Table 8:** SI of the study population according to age group in pre- and post-menopausal participants. The above table shows the SI on QUS among the study participants, categorized by their age and menopause status. SI: Stiffness Index; QUS: Quantitative Ultrasound.

Age	(n)	Post-menopausal		Pre-menopausal	
		Mean SI	SD	Mean SI	SD
21-30	19			87.63	24.93
31-40	78			85.35	13.35
41-50	118	78.46	10.56	84.04	14.68
51-60	97	74.27	14.90		
61-70	54	68.72	12.32		
71-80	4	62.75	7.46		

Table 9 depicts the correlation between the risk of osteoporosis and menopause in the study population. Out of the study participants (N=370), 200 had achieved menopause and 170 had not. Among the former group, 28 (14%) had osteoporosis, while among those who had not achieved menopause, osteoporosis was present in 6 (3.8%). There was a significant association between osteoporosis and menopause with a p-value <0.001 and an OR of 4.46 with a 95% CI.

**Table 9:** Association between osteoporosis and menopause in the study population. The above table compares the association between osteoporosis (T-score < -2.5) on QUS and menopause in the study population. There was a significant association between osteoporosis and menopause with a p-value <0.001 and an odds ratio of 4.46, with a 95% confidence interval. QUS: Quantitative ultrasound.

	Post-menopausal	Pre-menopausal	Total	Chi-square, p-value
Osteoporosis (T)	28	6	34	10.85, <0.001
No osteoporosis (T>-2.5)	172	164	336	
Total	200	170	370	

Table 10 shows the waist-to-hip ratio in the study population. Out of the 370 study participants, 34 patients had osteoporosis, 135 had osteopenia, and 201 had normal BMD. The mean WHR for osteoporotic study subjects was 0.823±0.035 cm, in osteopenia patients it was 0.821±0.028 cm, while in normal patients it was 0.817±0.028 cm. Thus, the mean WHR was lower in normal patients compared to osteoporotic subjects. However, there was no significant association (p-value >0.05) between the WHR and osteoporosis.

**Table 10:** Association of T-score on QUS with waist-to-hip ratio (N=370). F-statistics = 1.146, p-value = 0.3190. QUS: Quantitative ultrasound.

	T value	Mean waist-to-hip ratio	SD
Osteoporosis (34)		0.823	0.035
Osteopenia (135)	-2.5--1	0.821	0.028
Normal (201)	>-1	0.817	0.028
	Grand Total	0.819	0.028

## Discussion

QUS bone evaluation is a less expensive and more user-friendly alternative to the more conventional method of bone densitometry (measured BMD by x-ray absorptiometry). The two QUS parameters that are currently measured are BUA and SOS. The reported annual age-related changes in BUA and SOS for healthy women range from -0.27% to -1.62% and -0.06% to -0.19%, respectively. In terms of accuracy, BUA has a CV of 1.0-3.8% while SOS has a CV of 0.19-0.30%. It is believed that QUS mostly reflects BMD. However, according to a study, QUS also reflects trabecular orientation and other aspects of bone strength independently of BMD in both cross-sectional and prospective investigations. QUS may be a viable option for diagnosing osteoporotic individuals. A study by Heldan de Moura Castro C et al. measured three QUS parameters, BUA, SOS, and SI, in the right calcaneus of 352 healthy Caucasian Brazilian women aged between 20 and 84. Age was the most significant predictor for all QUS parameters (r = -0.49 for BUA, r = -0.66 for SOS, and r = -0.64 for SI). From values in young adulthood to those of women in their 90s, SI fell by around 41%, with approximately 76.4% of this loss occurring between the ages of 45 and 49 [18].

A recently created method for assessing fracture risk may evaluate both bone mass and architecture using QUS. Only a few studies have examined the relationship between the QUS parameter (SI) and menopausal status, despite multiple studies demonstrating that menopause is linked to an accelerated decrease in BMD. The mean SOS in the overall study population age group 21-30 was 1546.47±36.46, in age group 31-40 was 1546.34±28.42, in age 41-50 was 1540.32±30.16, in age 51-60 was 1529.70±25.68, and in age 61-70 was 1517.46±23.79. It is observed that as age advances, the SOS decreases. Among those who had achieved menopause, the SOS (in meters/s) was 1533.37±27.82 in the age group 41-50, 1529.70±25.68 in the age group 51-60, 1517.64±23.98 in group 61-70, and 1510.15±21.83 among the 71-80 age group. Thus, the mean SOS decreases as age advances in the group that has achieved menopause.

Similarly, in the other group where menopause has not been achieved, the mean±SD of the 21-30 age group is 1546.47±36.26, in age 31-40 is 1546.34±28.42, and in the 41-50 age group it has been 1544.76±30.94. Thus, a similar decrease in the SOS has been observed as age advances in those who have not achieved menopause. The mean BUA (dB/MHz) in the age group 21-30 was observed to be 111.96±24.05, in the age group 31-40 was 106.99±15.99, 41-50 was 105.90±10.71, 51-60 was 98.35±16.88, and 61-70 was 95.79±11.35. It is observed that as age advances the mean BUA decreases.

The mean T-score on QUS in the age group 21-30 is -0.71±1.58, which improves to -0.66±0.90 in the age group 31-40. However, thereafter the mean T-score decreases as age advances. This can also be observed in the figure earlier with the T-score decreasing as age advances. The mean T-score among those who had achieved menopause was -1.52±0.97, while the mean T-score among those who had not achieved menopause was -0.71±1.05. There was a significant difference in BMD based on menopause status. Out of the 370 study participants, 34 had menopause as indicated on the QUS. Among this group, 28 (14%) had osteoporosis, while for those who had not achieved menopause, osteoporosis was present in 6 (3.8%). There was a significant association between osteoporosis and menopause with a p-value <0.001.

The mean SI of the study participants was 78.77±15.62. It is observed that as the age increases, the mean SI of the study participants decreases. The mean SI among those who had achieved menopause was 73.53±13.68, while the mean SI among those who had not achieved menopause was 84.98±15.52. There was a significant difference between SI and menopause status. Among those who had achieved menopause, the SI was 78.46±10.56 in the age group 41-50, 74.27±14.90 in the age group 51-60, 68.72±12.32 in group 61-70, and 62.75±7.46 among the 71-80 age group. Thus, the mean SI decreases as age advances in the group that has achieved menopause.

Similarly in the other group where menopause has not been achieved, the mean±SD of the 21-30 age group is 87.63±24.93, in age 31-40 is 85.35±13.35, and in the 41-50 age group it is 84.04±14.68. Thus, a similar decrease in the SI has been observed as age advances in those who have not achieved menopause. In 19 nursing homes for the institutionalized elderly, Krieg MA et al. (1998) examined the effects of anthropometric parameters and biochemical markers of bone metabolism on QUS of bone. In 264 women who were 85 ± 7 (SD) years old and 103 males who were 81 ± 8 years old, QUS of the calcaneus and biochemical parameters were assessed. Age and BMI are strongly linked with BUA, according to QUS data (r = -0.297, 0.403, and -0.195, respectively) [19].

BUA is strongly and modestly linked with BMD at the lumbar spine, femoral neck, and radius midshaft, according to a study by Mészáros S et al. in males. However, at the aforementioned sites, SOS did not correlate with BMD [20]. BUA and SOS were substantially linked with BMD at several skeletal locations at the first screening and seven years later in a longitudinal study by Trimpou P et al., which included 80 Swedish women aged 53 to 73 years. Additionally, there was a substantial correlation between the changes in DXA and QUS readings throughout the course of the follow-up period [21].

In a study by Krieg MA et al. in males, an association between ultrasonography data and BMI (r = 0.326) was found. Age, BMI, biochemical markers, and QUS were examined in a multivariate analysis on women, but not on men due to their lesser number. Age, BMI, parathyroid hormone (PTH), and phosphate all accounted for 30% of the variation in BUA. Last but not least, QUS of bone evaluates bone characteristics that are affected, at least in part, by age, BMI, and secondary hyperparathyroidism brought on by vitamin D insufficiency [20]. Yoshimi I et al. (2001) studied 506 community-dwelling Japanese women between the ages of 40 and 89 for age-specific changes in SI and their relationships with age, BMI, and menopausal status. Of these, 459 women who had natural menopause had a mean age at menopause (standard deviation) of 49.4 (4.0) years. With advancing age, the SI considerably dropped. When compared to the 40-49 age group, the SI for those aged 80 to 89 was 40% lower. Between the ages of 40 and 49 and 50 to 59, the SI showed the highest reduction (15%) across neighboring ten-year age groups. The results show that menopause affects bone mass loss and causes degeneration in bone trabecular microstructure [22].

Adami S evaluated 6,811 postmenopausal women between the ages of 40 and 80 and 4,981 males between the ages of 60 and 80 from all areas of Italy to determine the impact of age and weight on calcaneal ultrasonography. He noted that age and weight had a significant impact on stiffness. A number of categorical characteristics that were controlled for age and weight were linked to stiffness as well. Both men and women were equally affected by the categorical and continuous factors that predicted stiffness [23]. The differences between calcaneal ultrasound measures and the ultrasonic quantitative index (QUI) in connection to menopause were investigated by López-Caudana AE et al. in Mexico. There were 862 women in it, ranging in age from 20 to 90. These women were included in a sequential sample for this cross-sectional study. She concluded that SOS in the calcaneus did not indicate menopausal alterations [24].

The impact of age, lifestyle characteristics, and body anthropometry on the calcaneal SOS (in meters/s) in a group of Malaysian males of various ages was investigated in a cross-sectional study by Chin KY et al. on 687 eligible individuals. He noted that although a difference was only identified in the older patients (p > 0.05), individuals with higher BMI had higher calcaneal SOS levels. Sedentary individuals reported lower calcaneal SOS readings than physically active individuals, although only middle-aged individuals showed a significant difference (p < 0.05). Age and physical activity score for all individuals were substantially (p < 0.05) linked with calcaneal SOS in young men, and height, BMI, and physical activity score in middle-aged and old men [25].

In order to further investigate the relationship between obesity and the calcaneus stiffness index (CSI), a measurement of bone density, Ali K et al. recruited 273 people for their community-based cross-sectional study in an Egyptian population. QUS was used to quantify the CSI, and the QUS T- and Z-scores of the non-dominant heel were also recorded. The average BMI was 29.7 (5.6) and the average age was 61 (11.9) years [26]. Even after adjusting for gender (P=0.041, O.R.=1.7), BMI was still substantially linked with SI in the older age group (P=0.049, O.R.=1.73). Older participants (55 years and above) exhibited considerably reduced bone density as determined by CSI compared to younger patients, and their BMI was strongly positively correlated with bone density.

Given the increase in QUS devices being used internationally, it is essential to develop methods for the clinical use of QUS. To determine the T-score threshold that would be appropriate to identify women at risk of osteoporosis using QUS, Frost conducted a study utilizing the WHO Study Group criteria for osteoporosis diagnosis to examine how T-scores change with age and the prevalence of the disease.

Weight, BMI, and age

As anthropometric parameters were analyzed, BMI was one of the parameters examined. Of our study participants, 213 (58%) had a normal BMI (18.5-24.9), followed by 85 (23%) who were underweight with a BMI <18.5. Pre-obese (BMI 25-29.9) women formed 57 (15%) of the study subjects, while obese class 1 (BMI 30-34.9) and obesity class 2 (BMI >35) were 13 (3%) and 2 (1%), respectively. The BMI of the study population was observed to be 21.62±4.21. Weight, BMI, and age showed the largest correlations with BMD values at the analyzed sites, according to a study by Soltani A et al. (2014) based on data collected from a referral center. Although age and BMD values are inversely associated at each of the study locations, there is a positive correlation between weight, height, and BMI and these BMD measures [27].

According to Knapp KM et al., body weight and weight fluctuations have an impact on the accuracy of DXA measurements of hip and spine density [28].

According to Saarelainen J et al., obesity (BMI more than 30 kg/m^2^) appears to delay the onset of osteopenia by five years in the spine and nine years at the hip (at the femoral neck). They proposed two explanations for this finding: the high baseline BMD of obese individuals or the way their greater fat mass affects X-ray attenuation [29].

Premenopausal women and adult males were the focus of a study by Zhao LJ et al. that was conducted among Caucasian and Chinese populations and independently examined. The subjects' mean ages were 27.2±4.5 years, and the range was 19.6-45.1 years. BMI was a reliable predictor of BMD, although among Caucasians, the standardized beta coefficients for the lumbar spine and femoral neck were only 0.23 and 0.25, respectively. On the other hand, fat mass and bone mass were found to be inversely correlated by Zhao LJ et al. The effects of mechanical stress from body weight on bone mass were controlled in this study [7].

Limitations

The participants were not concurrently studied by other diagnostic means. Hence, comparison with other databases should be interpreted with caution.

As the study was carried out in a rural population, we were not able to collect exact information about the age at menarche and at menopause, nor about the nutritional intake of the participants.

Due to the unavailability of a dual-energy X-ray absorptiometry (DEXA) scan, which is the gold standard for assessing bone mineral density, accurate data could not be collected.

## Conclusions

The study population falling under the categories of underweight and obese has been shown to have reduced BMD, whereas normal BMI is associated with normal BMD. Therefore, BMI and weight can be used to screen those at risk of osteoporosis and its associated problems. Also, as age advances, a decreasing trend is seen in SI, BUA, and SOS.

In the study population, post-menopausal women had a four times higher risk of osteoporosis than pre-menopausal women (OR=4.46). Thus, menopause also plays a role in influencing BMD in pre- and post-menopausal women.

Calcaneal QUS is potentially helpful as a pre-screening tool for the evaluation of osteoporosis, although it must be based on device-specific cut-offs that have been tested in the populations for which they are intended. Once population-specific interpretations are in place, pilot studies to explore the utilization of peripheral health workers in the community to screen for osteoporosis may be carried out.
